# piOxi database: a web resource of germline and somatic tissue piRNAs identified by chemical oxidation

**DOI:** 10.1093/database/baad096

**Published:** 2024-01-10

**Authors:** Kai Wang, Bambarendage P U Perera, Rachel K Morgan, Kimberley Sala-Hamrick, Viviana Geron, Laurie K Svoboda, Christopher Faulk, Dana C Dolinoy, Maureen A Sartor

**Affiliations:** Department of Environmental Health Sciences, School of Public Health, University of Michigan, 1415 Washington Heights, Ann Arbor, MI 48109, USA; Department of Environmental Health Sciences, School of Public Health, University of Michigan, 1415 Washington Heights, Ann Arbor, MI 48109, USA; Department of Environmental Health Sciences, School of Public Health, University of Michigan, 1415 Washington Heights, Ann Arbor, MI 48109, USA; Department of Environmental Health Sciences, School of Public Health, University of Michigan, 1415 Washington Heights, Ann Arbor, MI 48109, USA; Department of Environmental Health Sciences, School of Public Health, University of Michigan, 1415 Washington Heights, Ann Arbor, MI 48109, USA; Department of Pharmacology, School of Medicine, University of Michigan, 1150 W. Medical Center Drive, Ann Arbor, MI 48109, USA; Department of Animal Science, College of Food, Agricultural and Natural Resource Sciences, University of Minnesota, 1988 Fitch Avenue, Saint Paul, MN 55108, USA; Department of Environmental Health Sciences, School of Public Health, University of Michigan, 1415 Washington Heights, Ann Arbor, MI 48109, USA; Department of Nutritional Sciences, School of Public Health, University of Michigan, 1415 Washington Heights, Ann Arbor, MI 48109, USA; Department of Computational Medicine and Bioinformatics, School of Medicine, University of Michigan, 100 Washtenaw Ave, Ann Arbor, MI 48109, USA; Department of Computational Medicine and Bioinformatics, School of Medicine, University of Michigan, 100 Washtenaw Ave, Ann Arbor, MI 48109, USA; Department of Biostatistics, School of Public Health, University of Michigan, 1415 Washington Heights, Ann Arbor, MI 48109, USA

## Abstract

PIWI-interacting RNAs (piRNAs) are a class of small non-coding RNAs that are highly expressed and extensively studied from the germline. piRNAs associate with PIWI proteins to maintain DNA methylation for transposon silencing and transcriptional gene regulation for genomic stability. Mature germline piRNAs have distinct characteristics including a 24- to 32-nucleotide length and a 2ʹ-O-methylation signature at the 3ʹ end. Although recent studies have identified piRNAs in somatic tissues, they remain poorly characterized. For example, we recently demonstrated notable expression of piRNA in the murine soma, and while overall expression was lower than that of the germline, unique characteristics suggested tissue-specific functions of this class. While currently available databases commonly use length and association with PIWI proteins to identify piRNA, few have included a chemical oxidation method that detects piRNA based on its 3ʹ modification. This method leads to reproducible and rigorous data processing when coupled with next-generation sequencing and bioinformatics analysis. Here, we introduce piOxi DB, a user-friendly web resource that provides a comprehensive analysis of piRNA, generated exclusively through sodium periodate treatment of small RNA. The current version of piOxi DB includes 435 749 germline and 9828 somatic piRNA sequences robustly identified from *M. musculus, M. fascicularis* and *H. sapiens*. The database provides species- and tissue-specific data that are further analyzed according to chromosome location and correspondence to gene and repetitive elements. piOxi DB is an informative tool to assist broad research applications in the fields of RNA biology, cancer biology, environmental toxicology and beyond.

**Database URL:**  https://pioxidb.dcmb.med.umich.edu/

## Introduction

Small non-coding RNA (sncRNA) species, including microRNA (miRNA), small interfering RNA (siRNA) and PIWI-interacting RNA (piRNA), regulate a multitude of critical biological processes through epigenetic and biochemical mechanisms (1, 2). The piRNA class of sncRNAs has been extensively studied in the germline, where it is highly expressed and ranges in length from 24 to 32 nucleotides (3, 4). In addition to length, common features of germline piRNAs include the presence of a uridine signature at their 5ʹ ends; an adenosine signature at the 10th position, clustered within genomic regions and a stable, covalent 2ʹ-O-methylation modification at the 3ʹ end (1). piRNAs associate with PIWIL proteins to epigenetically silence transposable elements (TEs) by recruiting DNA methylation and regulate genomic integrity via transcriptional and post-transcriptional mechanisms (5–7). Since its discovery from *Drosophila melanogaster*, piRNA functions have been reported in multiple species including *Homo sapiens, Aplysia, Caenorhabditis elegans* and *Mus musculus*, with species-specific regulatory mechanisms for piRNA biogenesis (3, 8, 9). Although piRNA was once thought to be expressed and functionally exclusive to germline tissues, recent studies have identified piRNA-directed somatic functions distinct from the germline, including neuronal function, particularly long-term memory and tumor suppression (8, 10, 11). Somatic piRNA functions and mechanisms, however, remain poorly understood and require careful cataloging of species, tissue, sex, cell-type and age-specific piRNA data interrogation.

In contrast to piRNA, miRNAs have been extensively studied in association with several human pathologies such as cancer, cardiovascular disease, diabetes, neurological and immune disorders, as well as exposure to environmental chemicals including metals, endocrine disrupting chemicals and pollution (12–17). Given the close length ranges of piRNAs (20–36 nucleotides) and miRNAs (20–23 nucleotides), and the fact that they often occur in longer transcripts in groups or as preprocessed RNAs, distinguishing them based on read length alone is not precise. Furthermore, the exact length distribution of piRNAs in somatic tissues remains unclear, as most characterization has been performed on germline tissues (10). For example, our analysis of adult male murine somatic tissues from the three germ layers identified a high frequency of piRNA transcripts in a lower range of size distribution despite having consistent 2ʹ-O-methylation signatures at the 3ʹ end (10). Because piRNAs have been associated with human diseases and environmental exposures (18–22) and there is increasing focus on them, a comprehensive and highly specific database to confidently identify them is paramount. Thus, a piRNA database with detailed information on species, tissue, sex, cell-type, age and rigorous selection criteria is critical to assess disease or exposure-induced piRNA expression differences and develop piRNA-directed therapeutic approaches, including biomarker studies and epigenome editing technology for future biology, medicine and toxicology research (23, 24).

Current methods for piRNA detection include chromatography, small RNA size selection and RNA co-immunoprecipitation with PIWIL proteins, followed by next-generation sequencing. Although RNA co-immunoprecipitation methods have been considered the gold standard for piRNA detection, this method heavily relies on the purity, availability and quality of PIWIL antibodies, creating many challenges for research rigor and reproducibility. Alternatively, a small subset of publications has revealed that piRNA can be distinguished from other RNA species via sodium periodate treatment (10, 25, 26). This method was utilized to specifically select small RNAs containing the periodate-resistant 2ʹ-O-methylation signature at the 3ʹ end (a well-known characteristic of piRNA) and was incorporated with novel bioinformatics pipelines to detect piRNA (10). The periodate treatment of RNA chemically oxidizes piRNA via β-elimination, providing more robust and reproducible selection of piRNAs than previous methods. This method, coupled with next-generation sequencing and bioinformatics analysis, can test for multiple characteristics of piRNA including length, 5ʹ uridine signature, 10th position adenosine signature and clustering. Notably, the commonly used piRNA databases (piRBase, piRNAQuest, piRNABank and piRNA cluster database) classify piRNA primarily on length and association with PIWI interacting proteins only (27–30). To date, only piRBase includes sodium periodate–treated samples in their analyses.

Most of the publicly available databases have limited availability of piRNAs in somatic tissues, likely due to the lack of somatic piRNA research in the field. The functional implications of germline piRNA and fertility, along with evidence for sexual dimorphism in toxicology, diseases and ncRNA research, highlight the need for sex-specific characterization of piRNA in databases (31–34). It is worth noting that none of the aforementioned databases stratify data by sex, making it difficult to conduct sex-specific data interrogation for a given disease or exposure model. Some databases have limited data on tissue-specific piRNA information as well. As more sncRNA transcriptomic data become available for disease-associated biomarker identification, data interpretation and subsequent statistical analyses must be cautious of high heterogeneity and false-positive sequences included within piRNA databases (24). To address some of these key knowledge gaps, we introduce piOxi DB, a web resource of piRNA sequences and locations in multiple species, tissues and sexes that incorporates a comprehensive, rigorous analysis of piRNAs identified via sodium periodate treatment (https://pioxidb.dcmb.med.umich.edu/).

## Materials and Methods

### Sodium periodate treatment and data resources

**Figure 1.**
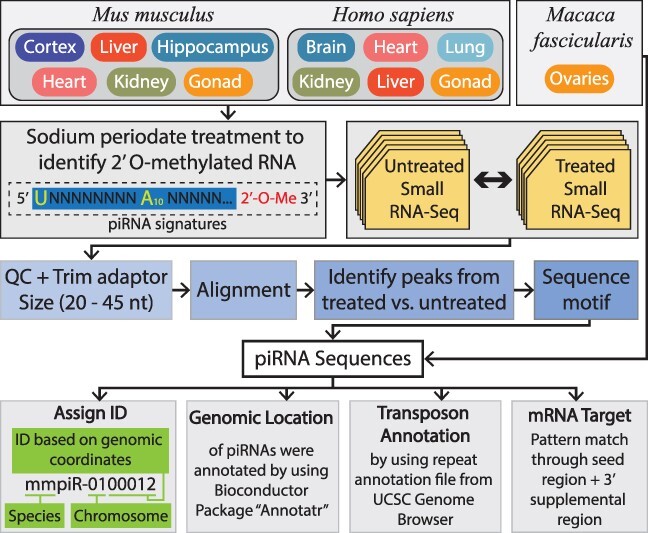
The top panel indicates the tissues from *M. musculus, M. fascicularis* and *H. sapiens* included within piOxi DB that were subjected to sodium periodate treatment. The second panel demonstrates that small RNA sequencing of the *M. musculus* and *H. sapiens* tissues was analyzed by comparing periodate-treated versus untreated samples. The third panel specifies how the data were processed using adapter trimming, alignment to the respective genome, identification of piRNA peaks between treated and untreated samples and determination of piRNA sequence motifs. The fourth panel designates the criteria used for piRNA identification assignment used for sequences and for genomic location, transposon annotation and mRNA targets used for annotations.

The sodium periodate treatment method (also known as β-elimination or periodate oxidation) specifically enriches for small RNAs containing a periodate-resistant 2ʹ-O-methylation signature, found at the 3ʹ end of mature piRNA transcripts. Since this modification prevents the base extension during sequencing, subsequent library preparation with PCR amplification can be utilized to select piRNAs (Figure 1). Mouse (*M. musculus*) tissue samples from wild-type non-agouti (93% genetically identical to the C57BL/6 J) background were used to derive small RNA (Qiagen AllPrep RNA isolation kits) with and without sodium periodate treatment. Libraries were prepared using the NEBnext small RNA kit (E7560S) and sequenced using the Illumina v4 HiSeq2500 with 50bp single-end reads with >35 million reads per sample. This process was previously detailed by Perera BP, *et al.* (10). Crab-eating macaque (*M. fascicularis*) data were obtained from a 2015 study (Figure 1) (25). The human (*H. sapiens*) data were generated from various tissues and sex by our group at the University of Michigan School of Public Health and Medical School with and without sodium periodate treatment. Human small RNAs were prepared using the Qiagen AllPrep RNA isolation kits; libraries were prepared using Takara SMARTer smRNA-seq library prep kit (Cat #635031) and sequenced using the Illumina NovaSeq S4 flowcell with 100bp single-end reads with >35 million reads per sample. All tissue samples were compared to detect piRNA transcripts by identifying sequences significantly enriched in the sodium periodate treatment samples compared to their matched control samples.

Alt text: Flowchart overview of the piOxi DB.

### Data processing

The human and mouse piRNA data were generated using the pipeline described by Perera *et al.* in 2019 (10). FastQC and MultiQC were used to assess the quality of raw sequencing data. Adapters and low-quality bases were trimmed by cutadapt (v1.18). After trimming, reads with lengths ≥10 and ≤45 bp were used for mapping. Bowtie2 (v2.2.9) with ‘end-to-end’ mode without mismatches was used for alignment in human genome (hg38) or mouse genome (mm10), and PePr (v1.1.24) was used for differential peak calling between periodate treated and control groups (35, 36). Peak calling was running on a RedHat Linux Server (v7.9) with parameters—shiftsize 0—windowsize 20—threshold 1E-3—peaktype sharp. An False Discovery Rate cutoff of <0.05 was used to select peaks significantly higher in periodate-treated groups and thus attributable to piRNA rather than other small RNA species. A unique piRNA ID was assigned to each significant peak based on the genomic coordinates (Figure 1). Bowtie2 was used to map the macaque-specific piRNA to the respective reference genome and the primary mapping was used to assign unique IDs. The Bioconductor package annotatr (v1.20.0) was used to find genomic features of all piRNAs. Transposon annotation files were downloaded from UCSC genome browser, and Bedtools (v2.26.0) was used to annotate piRNA sequences to repeat elements based on their genomic coordinates (36, 37). Messenger RNA (mRNA) sequences were also downloaded from the UCSC Genome Browser. Potential mRNA targets for piRNA were identified through an in-house Python script (https://github.com/sartorlab/piOxiDB-data-process-pipeline) to search for the seed and complementary regions of the piRNA sequences (38). All alignments without mismatches of macaque piRNAs were used to perform the genomic feature annotation and repeat annotations.

## Results

### Database design

**Figure 2.**
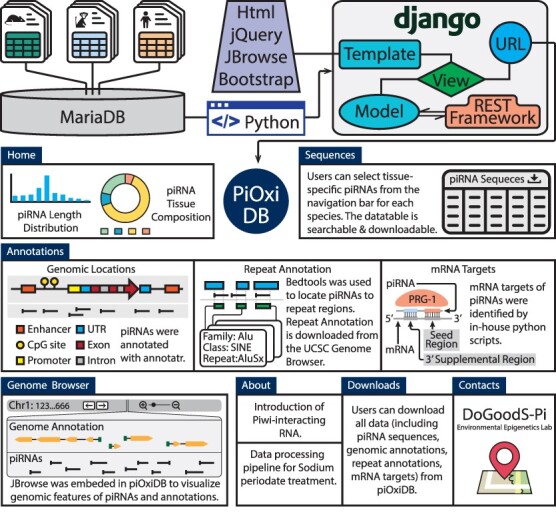
The piOxi DB website includes mouse, macaque and human piRNA data. Data were stored in MariaDB. Django with the REST framework was used to establish the webserver. JQuery, JBrowse and Bootstrap were used in implementing the frontend. The ‘Homepage’ includes a brief introduction and species-specific summary, including piRNA length distributions (histogram) and tissue compositions (pie chart). A representative table is included within the ‘Sequences’ that contains tissue-specific piRNA data for each species. The ‘Annotations’ tables indicate specific genomic locations, repeats and potential mRNA targets for the piRNA sequences found within piOxi DB. The ‘Browser’ function of piOxi DB allows the users to visualize the piRNA sequences of piOxi DB, relative to their corresponding genomic annotations. The ‘About’ section provides a brief introduction about piRNA, followed by ‘Downloads’ for data extrapolation and ‘Contacts’.

The *H. sapiens* data include the sex-specific (*N* = 5 males and 5 females) analysis of six tissues (brain, lung, heart, liver, kidney and gonads), collected during development (gestational days 90–105) (39). The *M. musculus* data include piRNA information derived from two male mice during adulthood (2–3 months of age), and include seven mouse tissues (cortex, hippocampus, liver, kidney, heart, ovaries and testis) representative of the three germ layers (10). The *M. fascicularis* data were ovary samples obtained from 2 macaques at 21 and 9 years of age (25). piOxi DB is a freely accessible database, where the data are stored and managed using MariaDB (v10.3.27). Django (v3.1.7) with the REST framework (v3.12.4) was used to establish the database. The CentOS Linux (v8) server, through the University of Michigan, was used to generate the website (https://pioxidb.dcmb.med.umich.edu/), which supports various browser types including Google Chrome, Safari and Firefox. NPM (v6.14.11) with React JS (v16.14.0) were utilized for embedding the react linear genome view from JBrowse2 in piOxi DB (Browser section) for genomic visualizations (40). The overall database design is shown in Figure 2.

Alt text: User-friendly features and utilities incorporated within piOxi DB.

### Bioinformatic pipeline for sodium periodate experiments

In piOxi DB, we provide a data processing pipeline to discover piRNAs from sodium periodate experiments. This pipeline is implemented in Python (version 3.8) and contains six steps: (i) FastQC and MultiQC; (ii) adapter trimming; (iii) FastQC after adapter trimming (including MultiQC); (iv) read length selection; (v) mapping and (vi) PePr peak calling. Users can download the pipeline from piOxi DB or through Github: https://github.com/sartorlab/piOxiDB-data-process-pipeline and use this pipeline to identify piRNA from any sodium periodate treated experiment for their own research experiments.

## User interface

The piOxi DB homepage is a user-friendly web interface that contains the main menu in a collapsible navigation bar on the left side of the website for easy access to processed piRNA data. The main components of the database are: (i) Home, (ii) Sequences, (iii) Annotations, (iv) Genome Browser, (v) About, (vi) Downloads and (vii) Contacts. The main menu items (b) and (c) are further categorized by animal species: Mouse (*M. musculus*), Macaque (*M. fascicularis*) and Human (*H. sapiens*). Each animal species incorporates a clickable drop-down function, with arrows indicating sub-categories that contain tissue-specific piRNA sequences for each species for (b), and specific piRNA annotations for (c). The footer of the website denotes that the data are copyrighted by the DoGoodS-Pi research group, including authors listed within the current manuscript.

### Home

The homepage sections are: (i) Introduction, (ii) Mouse piRNA Summary, (iii) Macaque piRNA Summary and (iv) Human piRNA Summary. The introduction section briefly explains the contents and features of the website. Sections (ii) through (iv) expand once clicked, providing literature references, sample information (species, tissues, sex and animal age), along with a summary of the piRNA length distribution (represented by a histogram: left panel) and piRNA tissue compositions (represented by a pie chart: right panel). The interactive animal piRNA length distribution histograms show piRNA transcript counts on the y-axis and transcript length in bp on the x-axis. The number of piRNA counts can be obtained by placing the cursor over the histogram bars. Similarly, the interactive piRNA tissue composition pie chart is color-coded by tissue type. The percentage and number of piRNA from each tissue can be found by placing the cursor over the respective components of the pie chart. These features allow the user to focus on interrogating the tissue-specificity of specific piRNA. The species and tissue-specific piRNA transcripts included in piOxi DB are summarized in Table 1.

**Table 1.** Number of unique piRNA sequences included within piOxi DB

Tissue	*M. musculus*	*M. fascicularis*	*H. sapiens*
Cortex	806		47 (whole brain)
Hippocampus	3 136		
Lung			4111
Heart	806		31
Liver	320		85
Kidney	365		121
Testis	24 047		446
Ovaries	5 754	403 941	1741

### Sequences

The Sequences page is categorized by animal species: mouse, macaque and human. As each category is expandable by selecting the species, the user is able to select a tissue of interest from the drop-down panel. For mouse, the drop-down panel includes cortex, hippocampus, liver, kidney, testis and ovary tissues, while macaque contains the ovary tissue. The human drop-down panel includes brain, lung, heart, liver, kidney and gonad (testis and ovary) tissues. Once the user clicks a chosen tissue type, a new page with detailed information about the references and navigation instructions appears on the top of the page, while the bottom of the page contains a table of tissue-specific piRNA sequencing data. The columns of each species and tissue-specific table contain the following information from left to right: (i) species information, (ii) primary tissue, (iii) sex (currently only for human data), (iv) piRNA identification (piRNA ID), a unique number generated by us to annotate each piRNA transcript, (v) piRNA sequence, (vi) piRNA sequence length, (vii) piRNA expression level (currently for mouse only), calculated according to information provided by the reference publication for mouse (10), (viii) *P*-value (currently for mouse and human) for each piRNA and (ix) signal value (currently for mouse and human) for each piRNA. The *P*-values and signal values were calculated from the PePr peak calling comparisons between periodate-treated and control groups. The piRNA ID is assigned based on the genomic coordinates for each piRNA sequence. The first two letters of the ID indicate the species information (‘mm’ for mouse, ‘mf’ for macaque and ‘hg’ for human), followed by the letters ‘piR’ to signify that it is a piRNA sequence. The first two numbers of the ID indicate chromosome number, followed by a sequence of five numbers assigned according to genomic coordinates. For instance, the piRNA ID ‘mmpiR-0100012’ represents a piRNA sequence found from the 12th piRNA position of mouse chromosome one. The up and down arrows next to each column title can be used to sort the data by column. A search box is made available towards the right side of the table to search for a specific piRNA sequence within the table, along with a ‘Download Table Content’ button above it to download and save the data in a user-friendly text (.txt) format.

### Annotations

The Annotations page is categorized by animal species (mouse, macaque and human), and each category can be expanded by subcategory (i) Genomic Location, (ii) Repeat Annotation and (iii) mRNA Targets. The user can select one of the subcategories from the drop-down panel for additional information on potential molecular mechanisms associated with the piRNA transcripts. Once chosen, a given subcategory leads to a new page with detailed information on published reference(s) from which the piRNA sequence annotation is derived, along with navigation instructions on the top of the page. Below the reference information is a clickable chromosome selection (represented via interactive ovals with colors) and a table of TE genomic locations, or potential mRNA targets for piRNA sequences, depending on the selected subcategory. The columns of table (i) contain the following information from left to right: Chrs (chromosome), piRNA ID, Seq Start (starting position of the piRNA sequence), Seq End (ending position of the piRNA sequence), Gene ID (NCBI gene identification), Gene Symbol (Gene common name), Type (piRNA alignment to 5ʹ UTR, promoter, exon, intron or 5ʹUTR regions), Anno Start (starting position of the annotated region of the genome), Anno End (ending position of the annotated region of the genome) and Strand (DNA orientation). Genomic coordinates for all annotations are from 5ʹ to 3ʹ. Thus, the genomic location subsection provides additional information relevant for piRNA biogenesis. Similarly, table (ii) columns contain the following information from left to right: Chrs, piRNA ID, Class/Family (the main class or family of TEs corresponding to piRNA), Repeat (the repeat types and subtypes of TEs), Repeat Start (starting position of the TE), Repeat End (ending position of the TE) and Strand. The Repeat Annotation provides information related to potential epigenetic regulation by piRNA, showing functional association to transposon silencing. The columns of table (iii) contain the following information from left to right: Chrs, piRNA ID, Gene Start (the mRNA starting position of the target mRNA), Gene End (the mRNA ending position of the target mRNA), Gene ID, Gene Symbol, Match (Reverse complement of the piRNA sequence that corresponds to the mRNA target) and Strand. Collectively, the mRNA target information may be used to extrapolate potential piRNA-directed transcriptional regulatory mechanisms. Tables (i) through (iii) can be reorganized using the up and down arrows next to each column title, and contain a search function to select specific piRNA sequences within each table. All data tables contain a search box towards their right side, along with a download box immediately above it, to extract and save the data in a user-friendly text (.txt) format.

### Genome browser

The genome browser page contains functions for custom visualization of the piRNA data. JBrowse2 software is embedded in piOxi DB to visualize multiple genome assemblies. The arrows and zoom functions allow the user to easily navigate across a given chromosome. The track selector can be opened to view the genome reference sequence, genome annotation, piRNA sequence, repeat annotation and mRNA targets for each species. In the mRNA targets section, all piRNAs that target each mRNA are shown in their corresponding locations.

### About

The About page provides a short overview to introduce piRNAs and provides background information and advantages of using piOxi DB. This section also includes a general description of the sodium periodate treatment method used to select for the piRNA compiled in the current database. We also include a visual representation of the computational pipelines used for small RNA sequencing analysis conducted to generate the piRNA sequences used in piOxi DB. In the future, all updates to this database will be included on the piOxi DB webpage.

### Downloads

Users can use the download feature for all piRNA sequence data available within piOxi DB by selecting the download boxes or icons. Downloadable data include all piRNA sequences from mouse, macaque and human, and their respective tissues, species-specific piRNA genomic locations, repeat annotations and mRNA targets. Downloaded data are provided in a user-friendly text (.txt) format that is available throughout the website.

### Contacts

The contact information page includes lead and senior author email contacts, along with links to laboratory websites and the physical address for the DoGoodS-Pi research group.

## Discussion

With accumulating data from germline and somatic piRNA sequencing experiments, piOxi DB was launched to provide accessible, comprehensive piRNA data specifically detected via sodium periodate treatment. The piOxi DB provides various classifications and annotations to facilitate the interpretation of piRNA expression based on species-, tissue- and sex-specific information. piRNAs play critical regulatory roles during development, disease states and other complex biological processes, suggesting their involvement in important tissue-specific functions in addition to transposon silencing in the germline (10, 41–43). For instance, piRNAs regulate neuronal differentiation via association with cold-shock domain proteins, cardiac circulatory system regeneration and genomic imprinting in the placenta (44–46).

As the future of piRNA research shifts to investigate somatic tissues and disease pathologies, more researchers are conducting high throughput sequencing and data mining approaches to discover piRNA biomarkers of disease. It is therefore imperative to create a publicly available piRNA database to accurately predict piRNA expression in somatic tissues (45). Currently available piRNA databases, including piRBase, piRNAQuest, piRNABank and piRNAclusterDB, are predominantly composed of germline tissues, with limited capacity for tissue- and sex-specific piRNA interrogation (24, 27–30). They also contain multiple piRNA detection methods such as RNA co-immunoprecipitation and small RNA sequencing approaches based on length, which introduce high sequence heterogeneity due in part to antibody purity and specificity. In contrast, the periodate treatment method (used for piRNA enrichment) coupled with bioinformatics approaches (10) (used to filter other ncRNA and false-positive data) provides a rigorous and reproducible technique for piRNA detection, laying the foundation to generate the comprehensive piRNA database, piOxi DB.

As more disease pathology and environmental exposure studies involve small RNA-seq experiments in their transcriptome analyses, piOxi DB will serve as a tool to identify and map potential environmental exposure and disease biomarkers, along with molecular mechanisms involving piRNA. These will advance the understanding of the piRNA pathways and elucidate piRNA functions associated with somatic tissues. PiOxi DB can be used in parallel with modifiable, publicly available computational tools and web resources to determine piRNA-related molecules and to identify piRNA-disease associations (47, 48). Our database has several user-friendly features that can be easily configured to assess omics data. The current database can be utilized alongside bioinformatics tools to identify differentially expressed piRNA sequences subjected to specific disease states, animal experiments, environmental exposures or chemical mixtures. We recommend the use of matched data types when using our database. For example, sncRNA sequencing data from 2-month-old male mouse brain samples from an experimental study can be compared with our piRNA sequence data from the mouse cortex or hippocampus. This ensures that the animal species, tissue and age are consistent with the user’s study design for rigorous and reproducible scientific research. Additionally, users can compare sncRNA sequencing data of interest from their treatment groups (derived from controls and environmental exposures or diseases), by using the genomic coordinates of piRNAs from piOxi DB (as a GTF file) and by determining differentially expressed piRNA via bioinformatics tools such as DESeq2 or edgeR (49, 50). The ‘Sequences’ section of our database allows users to search piRNAs by the sequence ID as well as sequence content. For example, if a user types ‘AAAAA’ into the search box, the output data table will include all piRNAs containing this substring in any region within a given piRNA sequence. Since piRNAs are sncRNAs and highly related to repeat sequences, they may also contain short, repeated adenines in their sequences (4). This feature enables users to detect any piRNA sequence based on sequence similarities. The ‘Annotation’ feature of piOxi DB can be used to identify critical information about piRNA biogenesis, potential TEs and mRNA targets that may be associated with the differentially expressed piRNA. This knowledge will, in turn, inform downstream mechanistic implications and future directions for investigative hypotheses. The ‘Genome Browser’ feature also allows for a customizable visual representation of the piRNA data by species, tissue and sex. Thus, piOxi DB will assist in broadening the understanding of epigenetic regulation and functionality of this emerging class of sncRNAs.

## Availability and future directions

The piOxi database will be regularly updated to incorporate more oxidized piRNA sequences and their respective annotations based on experimental evidence and data availability. Additional species-, tissue-, sex- and age-specific piRNA information will be available in the database as the data are reported. Data updates, new features and bug fixes will be detailed on Github (https://github.com/sartorlab/piOxiDB-data-process-pipeline).

## Conclusions

piRNA can be distinguished from other RNA species via sodium periodate treatment, which we utilized to select for its hallmark 2ʹ-O-methylation signature. We have compiled data from mouse, macaque and human somatic tissues to create the first piRNA web resource that exclusively identifies piRNA using periodate oxidation. The piOxi database contains small RNA sequencing data following sodium periodate treatment to generate detailed information on species-, tissue- and sex-specific piRNA-expressed sequences, along with annotations for piRNA genomic locations, TEs and mRNA targets. This database also provides genome mapping results and a summary of all available piRNA sequences. We have also integrated user-friendly search and download functions within the database for broad research applications in the fields of RNA biology, cancer biology, environmental toxicology and beyond.

## Data Availability

piOxi DB is publicly available at https://pioxidb.dcmb.med.umich.edu/.

## References

[1] Zuo L.J. , Wang Z.R., Tan Y.L. et al. (2016) piRNAs and their functions in the brain. *Int. J. Human Genet.*, 16, 53–60. 27512315 10.1080/09723757.2016.11886278 PMC4976825

[2] Mani S.R. and Juliano C.E. (2013) Untangling the web: the diverse functions of the PIWI/piRNA pathway. *Mol. Reprod. Dev.*, 80, 632–664. 23712694 10.1002/mrd.22195 PMC4234069

[3] Lin H. (2007) piRNAs in the germ line. *Science*, 316, 397. 10.1126/science.1137543 17446387

[4] Aravin A.A. , Sachidanandam R., Girard A. et al. (2007) Developmentally regulated piRNA clusters implicate MILI in transposon control. *Science*, 316, 744–747. 17446352 10.1126/science.1142612

[5] Carmell M.A. , Girard A., van de Kant H.J.G. et al. (2007) MIWI2 is essential for spermatogenesis and repression of transposons in the mouse male germline. *Dev. Cell*, 12, 503–514. 17395546 10.1016/j.devcel.2007.03.001

[6] Czech B. and Hannon G.J. (2016) One loop to rule them all: the ping-pong cycle and piRNA-guided silencing. *Trends Biochem. Sci.*, 41, 324–337. 26810602 10.1016/j.tibs.2015.12.008 PMC4819955

[7] Grivna S.T. , Beyret E., Wang Z. et al. (2006) A novel class of small RNAs in mouse spermatogenic cells. *Genes Dev.*, 20, 1709–1714. 16766680 10.1101/gad.1434406 PMC1522066

[8] Rajasethupathy P. , Antonov I., Sheridan R. et al. (2012) A role for neuronal piRNAs in the epigenetic control of memory-related synaptic plasticity. *Cell*, 149, 693–707. 22541438 10.1016/j.cell.2012.02.057 PMC3442366

[9] Parhad S.S. and Theurkauf W.E. (2019) Rapid evolution and conserved function of the piRNA pathway. *Open Biol.*, 9, 180181. 10.1098/rsob.180181 PMC6367137 30958115

[10] Perera B.P.U. , Tsai Z.T.-Y., Colwell M.L. et al. (2019) Somatic expression of piRNA and associated machinery in the mouse identifies short, tissue-specific piRNA. *Epigenetics*, 14, 504–521. 30955436 10.1080/15592294.2019.1600389 PMC6557559

[11] Liu Y. , Dou M., Song X. et al. (2019) The emerging role of the piRNA/piwi complex in cancer. *Mol. Cancer*, 18, 123. 10.1186/s12943-019-1052-9 PMC6688334 31399034

[12] Zhou S.S. , Jin J.-P., Wang J.-Q. et al. (2018) miRNAS in cardiovascular diseases: potential biomarkers, therapeutic targets and challenges. *Acta Pharmacol. Sin.*, 39, 1073–1084. 29877320 10.1038/aps.2018.30 PMC6289363

[13] Lee Y.S. and Dutta A. (2009) MicroRNAs in cancer. *Annu. Rev. Pathol.*, 4, 199–227. 18817506 10.1146/annurev.pathol.4.110807.092222 PMC2769253

[14] Ferragut Cardoso A.P. , Udoh K.T. and States J.C. (2020) Arsenic-induced changes in miRNA expression in cancer and other diseases. *Toxicol. Appl. Pharmacol.*, 409, 115306. 10.1016/j.taap.2020.115306 PMC7772821 33127375

[15] Bommarito P.A. , Martin E. and Fry R.C. (2017) Effects of prenatal exposure to endocrine disruptors and toxic metals on the fetal epigenome. *Epigenomics*, 9, 333–350. 28234024 10.2217/epi-2016-0112 PMC5827796

[16] Li X. , Haberzettl P., Conklin D.J. et al. (2021) Exposure to fine particulate matter air pollution alters mRNA and miRNA expression in bone marrow-derived endothelial progenitor cells from mice. *Genes*, 12, 1058. 10.3390/genes12071058 PMC8307414 34356074

[17] Zhang L. , Lu Q. and Chang C. (2020) Epigenetics in health and disease. *Adv. Exp. Med. Biol.*, 1253, 3–55. 32445090 10.1007/978-981-15-3449-2_1

[18] Xin J. , Du M., Jiang X. et al. (2021) Systematic evaluation of the effects of genetic variants on PIWI-interacting RNA expression across 33 cancer types. *Nucleic Acids Res.*, 49, 90–97. 33330918 10.1093/nar/gkaa1190 PMC7797066

[19] Pierouli K. , Papakonstantinou E., Papageorgiou L. et al. (2023) Role of non-coding RNAs as biomarkers and the application of omics technologies in Alzheimer’s disease (review). *Int. J. Mol. Med.*, 51, 1–11. 36453246 10.3892/ijmm.2022.5208 PMC9747195

[20] Corsello T. , Kudlicki A.S., Garofalo R.P. et al. (2019) Cigarette smoke condensate exposure changes RNA content of extracellular vesicles released from small airway epithelial cells. *Cells*, 8, 1652. 10.3390/cells8121652 PMC6953119 31861112

[21] Mias G.I. , Singh V.V., Rogers L.R.K. et al. (2021) Longitudinal saliva omics responses to immune perturbation: a case study. *Sci. Rep.*, 11, 710. 10.1038/s41598-020-80605-6 PMC7804305 33436912

[22] Perera B.P.U. , Svoboda L. and Dolinoy D.C. (2019) Genomic tools for environmental epigenetics and implications for public health. *Curr. Opin. Toxicol.*, 18, 27–33. 31763499 10.1016/j.cotox.2019.02.008 PMC6874218

[23] Perera B.P. , Faulk C., Svoboda L.K. et al. (2019) The role of environmental exposures and the epigenome in helath and disease. *Environ. Mol. Mutagen.*, 61, 176–192. 31177562 10.1002/em.22311 PMC7252203

[24] Perera B.P.U. , Morgan R.K., Polemi K.M. et al. (2022) PIWI-interacting RNA (piRNA) and epigenetic editing in environmental health sciences. *Curr. Environ. Health Rep.*, 9, 650–660. 35917009 10.1007/s40572-022-00372-6

[25] Roovers E.F. , Rosenkranz D., Mahdipour M. et al. (2015) Piwi proteins and piRNAs in mammalian oocytes and early embryos. *Cell Rep.*, 10, 2069–2082. 25818294 10.1016/j.celrep.2015.02.062

[26] Kawano M. , Kawaji H., Grandjean V. et al. (2012) Novel small noncoding RNAs in mouse spermatozoa, zygotes and early embryos. *PLoS One*, 7, e44542. 10.1371/journal.pone.0044542 PMC3440372 22984523

[27] Wang J. , Shi Y., Zhou H. et al. (2022) piRBase: integrating piRNA annotation in all aspects. *Nucleic Acids Res.*, 50, D265–D272. 34871445 10.1093/nar/gkab1012 PMC8728152

[28] Ghosh B. , Sarkar A., Mondal S. et al. (2022) piRNAQuest V.2: an updated resource for searching through the piRNAome of multiple species. *RNA Biol.*, 19, 12–25. 34965192 10.1080/15476286.2021.2010960 PMC8786328

[29] Sai Lakshmi S. and Agrawal S. (2008) piRNABank: a web resource on classified and clustered Piwi-interacting RNAs. *Nucleic Acids Res.*, 36, D173–D177. 17881367 10.1093/nar/gkm696 PMC2238943

[30] Rosenkranz D. , Zischler H. and Gebert D. (2022) piRNAclusterDB 2.0: update and expansion of the piRNA cluster database. *Nucleic Acids Res.*, 50, D259–D264. 34302483 10.1093/nar/gkab622 PMC8728273

[31] Burgos M. , Hurtado A., Jiménez R. et al. (2021) Non-coding RNAs: lncRNAs, miRNAs, and piRNAs in sexual development. *Sex Dev.*, 15, 335–350. 34614501 10.1159/000519237

[32] Liang X. , Feswick A., Simmons D. et al. (2018) Environmental toxicology and omics: a question of sex. *J. Proteomics*, 172, 152–164. 29037750 10.1016/j.jprot.2017.09.010

[33] Vaura F. , Palmu J., Aittokallio J. et al. (2022) Genetic, molecular, and cellular determinants of sex-specific cardiovascular traits. *Circ. Res.*, 130, 611–631. 35175841 10.1161/CIRCRESAHA.121.319891

[34] Svoboda L.K. , Neier K., Wang K. et al. (2021) Tissue and sex-specific programming of DNA methylation by perinatal lead exposure: implications for environmental epigenetics studies. *Epigenetics*, 16, 1102–1122. 33164632 10.1080/15592294.2020.1841872 PMC8510611

[35] Langmead B. and Salzberg S.L. (2012) Fast gapped-read alignment with Bowtie 2. *Nat. Methods*, 9, 357–359. 22388286 10.1038/nmeth.1923 PMC3322381

[36] Zhang Y. , Lin Y.H., Johnson T.D. et al. (2014) PePr: a peak-calling prioritization pipeline to identify consistent or differential peaks from replicated ChIP-Seq data. *Bioinformatics*, 30, 2568–2575. 24894502 10.1093/bioinformatics/btu372 PMC4155259

[37] Quinlan A.R. and Hall I.M. (2010) BEDTools: a flexible suite of utilities for comparing genomic features. *Bioinformatics*, 26, 841–842. 20110278 10.1093/bioinformatics/btq033 PMC2832824

[38] Zhang D. , Tu S., Stubna M. et al. (2018) The piRNA targeting rules and the resistance to piRNA silencing in endogenous genes. *Science*, 359, 587–592. 29420292 10.1126/science.aao2840 PMC5939965

[39] Morgan R. (2023) Investigating the impact of lead exposure on epigenetic regulation during neural differentiation and neurodevelopment: focus on piRNA and transposable element regulation. *Ph.D. Thesis*. University of Michigan.

[40] Diesh C. , Stevens G.J., Xie P. et al. (2023) JBrowse 2: a modular genome browser with views of synteny and structural variation. *Genome Biol.*, 24, 74. 10.1186/s13059-023-02914-z PMC10108523 37069644

[41] Sellitto A. , Geles K., D’Agostino Y. et al. (2019) Molecular and functional characterization of the somatic PIWIL1/piRNA pathway in colorectal cancer cells. *Cells*, 8, 1390. 10.3390/cells8111390 PMC6912267 31694219

[42] Huang S. , Ichikawa Y., Igarashi Y. et al. (2019) Piwi-interacting RNA (piRNA) expression patterns in pearl oyster (Pinctada fucata) somatic tissues. *Sci. Rep.*, 9, 247. 10.1038/s41598-018-36726-0 PMC6342924 30670741

[43] Song J. , Liu J., Schnakenberg S.L. et al. (2014) Variation in piRNA and transposable element content in strains of Drosophila melanogaster. *Genome Biol. Evol.*, 6, 2786–2798. 25267446 10.1093/gbe/evu217 PMC4224344

[44] Subhramanyam C.S. , Cao Q., Wang C. et al. (2022) piRNAs interact with cold-shock domain-containing RNA binding proteins and regulate neuronal gene expression during differentiation. *Mol. Neurobiol.*, 59, 1285–1300. 34982407 10.1007/s12035-021-02678-2

[45] Zhou Y. , Fang Y., Dai C. et al. (2021) PiRNA pathway in the cardiovascular system: a novel regulator of cardiac differentiation, repair and regeneration. *J. Mol. Med. (Berl)*, 99, 1681–1690. 34533602 10.1007/s00109-021-02132-9

[46] Martinez V.D. , Sage A.P., Minatel B.C. et al. (2021) Human placental piwi-interacting RNA transcriptome is characterized by expression from the DLK1-DIO3 imprinted region. *Sci. Rep.*, 11, 14981. 10.1038/s41598-021-93885-3 PMC8298716 34294738

[47] Liu Y. , Li A., Xie G. et al. (2021) Computational methods and online resources for identification of piRNA-related molecules. *Interdiscip. Sci.*, 13, 176–191. 33886096 10.1007/s12539-021-00428-5

[48] Ali S.D. , Tayara H. and Chong K.T. (2022) Identification of piRNA disease associations using deep learning. *Comput. Struct. Biotechnol. J.*, 20, 1208–1217. 35317234 10.1016/j.csbj.2022.02.026 PMC8908038

[49] Love M.I. , Huber W. and Anders S. (2014) Moderated estimation of fold change and dispersion for RNA-seq data with DESeq2. *Genome Biol.*, 15, 550. 10.1186/s13059-014-0550-8 PMC4302049 25516281

[50] Robinson M.D. , McCarthy D.J. and Smyth G.K. (2010) edgeR: a bioconductor package for differential expression analysis of digital gene expression data. *Bioinformatics*, 26, 139–140. 19910308 10.1093/bioinformatics/btp616 PMC2796818

